# Mometasone furoate nasal spray: a systematic review

**DOI:** 10.1186/s40248-016-0054-3

**Published:** 2016-05-02

**Authors:** Desiderio Passali, Maria Carla Spinosi, Anna Crisanti, Luisa Maria Bellussi

**Affiliations:** University of Siena- ENT Clinic, Siena, Italy; Department of Experimental Medicine and Surgery, University of Rome Tor Vergata, Rome, Italy

**Keywords:** Adenoid hypertrophy, Allergic rhinitis, Asthma, Mometasone furoate, Nasal polyposis, Rhinosinusitis

## Abstract

The inflammatory diseases of the nose, rhino-pharynx and paranasal sinuses (allergic and non allergic rhinitis, NARES; rhinosinusitis with/without nasal polyposis, adenoidal hypertrophy with/without middle ear involvement) clinically manifest themselves with symptoms and complications severely affecting quality of life and health care expenditure.

Intranasal administration of corticosteroids, being fast, simple, and not requiring cooperation, is the preferred way to treat the patients, to optimize their quality of life, at the same time minimizing the risk of exacerbations and complications.

Among the different topical steroids available on the market, we performed a comparative analysis in terms of effectiveness and safety between mometasone furoate (MF) and its main competitors.

Searching through Pub Med and Google Scholar and using as entries “mometasone furoate”, “rhinitis”, “sinusitis”, “asthma”, “polyposis”, “otitis media with effusion”, and “adenoid hypertrophy” we found 344 articles, 300 of which met the eligibility criteria.

Taking into account relevance and date of publication, a sample of 40 articles was considered for the review.

MF effectiveness for treatment and/or prophylaxis of nasal symptoms in seasonal and perennial allergic rhinitis has been fully established with a level of evidence Ia.

Even though it has not been assessed for MF in particular, topical steroids are the most appropriate treatment in mixed rhinitis and NARES.

In acute rhinosinusitis (ARS) evidences support their use as mono-therapy or as adjuvant to antibiotics for reducing the recurrence rate, and decrease the usage of related prescriptions and medical consultations.

In chronic rhinosinusitis (CRS) with Nasal polyposis, MF reduces polyps size, nasal congestion, improves quality of life and sense of smell and it is also effective in the treatment of daytime cough.

The topical use of MF has great efficacy in the management of adenoidal hypertrophy and otitis media of atopic children.

As regards the safety, MF has demonstrated an excellent safety profile: pregnant women can safely use it; no systemic effects on growth velocity and adrenal suppression have been shown; no changes in epithelial thickness or atrophy have been observed after long term administration of the drug.

**Conclusions:** MF has been demonstrated to be effective in the treatment of the inflammatory diseases of the nose and paranasal sinuses; when compared to its competitors it shows a greater symptom control; it is a reliable treatment in the long term thanks not only to its proven efficacy, but also to its safety being on the market since more than 17 years.

## Background

Rhinitis is a common inflammation of the nasal membrane of the nose, caused by viruses, bacteria, and other irritants as well as allergens. Even if it is usually associated with inflammation, some forms of rhinitis, such as vasomotor or athrophic rhinitis, are not predominantly inflammatory.

Chronic rhinosinusitis is an inflammatory condition of the nose and paranasal sinuses defined by: presence of two or more symptoms one of which should be either nasal blockage/obstruction/congestion or nasal discharge (anterior/posterior nasal drip) +/- facial pain/pressure; +/- reduction or loss of smell in adults (+/- cough in children) for ≥ 12 weeks (EPOS, 2012).

The persistent inflammatory condition of the nose and paranasal sinuses clinically manifests itself with many symptoms and complications, such as nasal congestion, sneezing, coughing, headaches, rhinorrhea (anterior or posterior), itching, malaise, pain, and fatigue. Those afflictions are frequently accompanied by symptoms involving the eyes, ears and throat and combined together, they all severely affect the quality of life.

Enlarged adenoids and infection of adenoid tissue may lead to the obstruction of the breathing patterns in children, causing apnea during sleep and contibuting to recurring sinusitis and persistent middle ear disease.

Rhinitis, as well as nasal congestion, may also lead to airway obstruction and an increased number of sleep microarousals, both in children and adults. Sleep disturbances can detrimentally affect daytime energy levels, mood, and, consequently, determine daytime fatigue [1].

Combined together, these afflictions account for 1 to 2 % of total physician consultations and are associated with large health care expenditure.

## Main text

Appropriate use of medical therapies is necessary to optimize patient quality of life and daily functioning and minimize the risk of acute inflammatory exacerbations and complications.

The most common therapies for the aforementioned diseases are topical nasal sprays which could be natural or pharmaceutical products. Intranasal route is generally preferred by both patients and doctors: it is fast, simple, does not require cooperation, the drug assimilation is direct and quick and compliance to therapy is high. Among these sprays, corticosteroid topical application is undoubtedly the most widely used and most effective [2, 3] although minor side effects could be reported (nose bleeding, dryness, crusting).

Glucocorticosteroids are used in upper airway diseases: allergic and non allergic rhinitis, particularly non allergic rhinitis with eosinophilia syndrome (NARES), acute and chronic rhinosinusitis, with and without nasal polyps, adenoid hypertrophy with or without middle era disease.

There are many other possible therapies, either medical or surgical, and especially for allergic rhinitis management many different therapeutical strategies are available.

Effective measures include allergen avoidance (pollens, fungi, dust), mite-proof covers, air filters and nasal irrigation, and the best results are obtained when combined with intranasal steroids. Other pharmacological options should be taken into account if those measures are not effective, and they include antihistamine drugs, that could be taken orally or nasally, pseudoephedrine, cromolyn, leukotriene receptor antagonists.

Thus far, evidence suggests that intranasal corticosteroids produce greater relief of nasal symptoms than topical antihistamines (H1 receptor antagonists), even if no difference in ocular symptoms has been reported [4].

Long-term tolerance to allergens can be induced by immunotherapy, but the desensitiziation therapy is often considered to be quite expensive [5]. Moreover, the capacity of sublingual allergen immunotherapy (SLIT) to provide effective symptom relief in seasonal allergic rhinitis has also been questioned. It has been reported that SLIT tablets had a greater clinical impact than second-generation antihistamines and montelukast, but when compared to nasal corticosteroids, the beneficial effects were the same [6].

Alternative treatments such as acupuncture and homeopathy, even if quite popular, are not supported by any evidence.

Intranasal corticosteroids may be useful in the treatment of some forms of non-allergic rhinitis and should be an appropriate choice for mixed rhinitis. Evidence [7] declares them to be useful for the treatment of non allergic rhinitis with eosonophilia syndrome.

Also for chronic (CRS) and acute (ARS) rhinosinusitis various topical therapies are available: saline solutions, antibiotics, corticosteroids, and antifungals. Topical saline and corticosteroids should be considered as the first line of therapy for CRS [8] and evidence supports its use as a monotherapy or as an adjuvant therapy to antibiotics in ARS [9].

Corticosteroid nasal sprays include: beclometasone diproprionate, budesonide, ciclesonide, flunisolide, fluticasone furoate, fluticasone proprionate, triamcinolone acetonide, and mometasone furoate.

With so many different sprays available in the market, many authors decided to compare efficacy, side-effect profile and relative cost of each product, to assess which - and if - one is the best [2]. Results have too often been controversial and there is no clear evidence in favour of one or the other. Thus far it has been established that all the sprays have a similar side-effect profile, and the most significant differences might be related to patient's personal preference for each product sensory attributes [10–12] and costs.

Mometasone furoate (MF) is used in the treatment of rhinitis and rhinosinusitis as well as asthma [13], inflammatory skin disorders and penile phimosis. Evidence suggests that its usage improves symptoms related to adenoid hypertrophy, too.

To assess MF nasal spray effectiveness and safety we decided to analyze the scientific publications related to this molecule and to perform a comparative analysis between MF and its main intranasal competitors.

Our work consisted in searching through Pub Med and Google Scholar, using as entries: “mometasone furoate”, “rhinitis”, “sinusitis”, “asthma”, “polyposis”, “pediatric OSAS”, “otitis media with effusion” and “adenoid hypertrophy.”

The database search revealed 344 articles. Out of these, 300 met eligibility criteria and were assessed independently by two authors for further evaluation. A sample of 40 articles was considered for this review, taking into account relevance and date of publication.

We then proceded to evaluate mometasone furoate nasal spray efficacy, safety, and cost-effectivenes.

In 1998, mometasone furoate was introduced as a nasal spray. MF has been largely successful since it was first sold. Its dosage is 2 sprays/nostril once a day (50 mcg in each spray) and the dosage for children is halved to 1 spray for each nostril daily. Evidence [14–16] shows that - at least for asthma maintenance - 1spray daily should be enough (thus diminishing both costs and side effects).

To obtain the best results, as most topical intranasal corticosteroids, it is strongly suggested to blow the nose to clear the passageway before the application or perform a nasal irrigation, and to avoid sneezing or blowing the nose right after spraying.

MF usage for treatment and/or prophylaxis of the nasal symptoms of seasonal allergic rhinitis and perennial allergic rhinitis has been fully established and a level of evidence Ia (Centre for Evidence Based Medicine, Oxford 2009) has been provided [17, 18].

Intranasal corticosteroid therapy also has a beneficial effect in relieving eye symptoms in allergic conjunctivitis, similar to oral or intranasal antihistamines [19–21] according to reviews.

Even though it has not been clearly assessed for MF in particular, its usage should improve other forms of rhinitis, like mixed forms or NARES.

Compared with antibiotics monotherapy, using MF nasal spray for initial ARS treatment, alone or combined with an antibiotic, has been demonstrated to reduce the recurrence rate and decrease the usage of related prescriptions and medical consultations [22].

As regards the treatment of nasal polyposis, MF, if administered daily, reduces polyp size and the nasal congestion, improving quality of life and sense of smell, with no unusual or unexpected adverse events [23–25].

Furthermore, regarding other minor complaints associated with rhinitis and rhinosinusitis, mometasone furoate nasal spray has been shown to be safe, effective and well tolerated in the treatment of daytime cough, too [26].

In 1998, Minshall et al. [27] evaluated patients before and after treatment with MF nasal spray. Through nasal biopsies, they assessed that long-term administration of the drug attenuates the inflammatory process decreasing the extent of inflammatory cell infiltration (especially eosinophils) and did not determine changes in epithelial thickness or atrophy.

It has also been well established that the topical use of MF in the pediatric population shows great efficacy for management of adenoidal hypertophy [28] and otitis media with effusion [29], despite the concomitant presence of atopy [30]. Lastly, as reported in a recent cost-effectiveness analysis [31] when compared to beclomethasone diproprionate, the therapy with mometasone furoate for treating children suffering from allergic rhinitis showed a greater improvement, better efficacy, safety, and lower total treatment cost.

As regards the safety of the drug, the same results were obtained when administered in children for nasal polyps, even at double the recommended pediatric dosage for allergic rhinitis [32].

Pregnant women can safely use intranasal steroids. The sprays work only in the nasal passageway and the medicine does not affect other parts of the body unless too much is used [3].

Until now, despite parents apprehension, topical application of MF - respecting the recommended dosage - has not shown systemic effects. Many studies have been conducted to investigate its effects on growth velocity and its potential to cause adrenal suppression [33, 34]. Thus far, no significant difference has been reported and the current guidelines for asthma recommend inhaled corticosteroids for the control of mild to severe persistent asthma in adults as well as adolescents.

MF dry powder inhaler has been demonstrated to have an excellent safety and efficacy profile, and during post-marketing surveillance and in clinical trials no significant adverse side effects have been reported. In addition, its simple use seems to improve asthma management by addressing issues that generally inhibit proper adherence to therapy [35–38]. These considerations for the usage of MF in asthma could be extended to MF administered via the intranasal route.

Each steroid preparation comes with patient instructions for safe and effective use. For best results, the medication should be used following this direction carefully and according to medical advice. Adherence to long-term therapy is essential to have the desired results. In fact, intermittent therapy may not guarantee the same benefits.This is even more true in the case of a chronic disease. Moreover, non-adherence to treatment may also require that, if an acute attack occurs, a higher dose of medication would be required, and possibly even asteroid molecule orally or intramuscularly administered.

The large quantity of different preparations available could also allow the doctor - in case of molecules with the same efficacy, dosage and quality of the dispenser - to choose the most convenient for the patient, taking into account the expenses related with long term use.

Expenses are halved when analyzing children (up to 11 years of age), due to the fact that the recommmended dosages are of one spray for each nostril once daily.

Many preference evaluation studies have been conducted to assess MF sensory attributes and establish patients’ preference. Patients’ choice/preference might contribute to enhanced treatment outcomes, since it could improve adherence to treatment [10–12]. Results were not always in favour of MF, but Meltzer et al. [12] reported it to be more agreeable according to users.

Last but not least, it is a fact that all steroids suppress gene expression of factors responsible for generating and supporting inflammatory processes but furoates earn special attention as their lateral furoate ester chain makes the molecules highly lipophilic. Thus, the moleculas are easily absorbed by mucous membranes, epithelium and cell membrane phospholipids. This minimizes their general action and maximizes local action [39].

## Conclusions

MF has been demonstrated to be safe, effective and, if compared to its competitors, it shows symptom control greater than the other products in the market. Moreover, even if its marketing might classify it as an “old” molecule, it is also true that over 17 years of proven efficacy not only define it as reliable in the long term, but make MF absolutely safe as well.

## References

[1] Craig TJ, Teets S, Lehman EB, Chinchilli VM, Zwillich C (1998). Nasal congestion secondary to allergic rhinitis as a cause of sleep disturbance and daytime fatigue and the response to topical nasal corticosteroids. J Allergy Clin Immunol.

[2] Waddell AN, Patel SK, Toma AG, Maw AR (2003). Intranasal steroid sprays in the treatment of rhinitis: is one better than another?. J Laryngol Otol.

[3] Wallace DV, Dykewicz MS, Bernstein DI, Blessing-Moore J, Cox L, Khan DA (2008). The diagnosis and management of rhinitis: an updated practice parameter. J Allergy Clin Immunol.

[4] Yáñez A, Rodrigo GJ (2002). Intranasal corticosteroids versus topical H1 receptor antagonists for the treatment of allergic rhinitis: a systematic review with meta-analysis. Ann Allergy Asthma Immunol.

[5] Bachert C, Vestenbaek U, Christensen J, Griffiths UK, Poulsen PB (2007). Cost-effectiveness of grass allergen tablet (GRAZAX®) for the prevention of seasonal grass pollen induced rhinoconjunctivitis – a Northern European perspective. Clin Exp Allergy.

[6] Devillier P, Dreyfus JF, Demoly P, Calderón MA (2014). A meta-analysis of sublingual allergen immunotherapy and pharmacotherapy in pollen-induced seasonal allergic rhinoconjunctivitis. BMC Med.

[7] Webb DR, Meltzer EO, Finn AF, Rickard KA, Pepsin PJ, Westlund R (2002). Intranasal fluticasone propionate is effective for perennial nonallergic rhinitis with or without eosinophilia. Ann Allergy Asthma Immunol.

[8] Huang A, Govindaraj S (2013). Topical therapy in the management of chronic rhinosinusitis. Curr Opin Otolaryngol Head Neck Surg.

[9] Zalmanovici Trestioreanu A, Yaphe J (2013). Intranasal steroids for acute sinusitis. Cochrane Database Syst Rev.

[10] Meltzer EO, Bardelas J, Goldsobel A, Kaiser H (2005). A preference evaluation study comparing the sensory attributes of mometasone furoate and fluticasone propionate nasal sprays by patients with allergic rhinitis. Treat Respir Med.

[11] Yonezaki M, Akiyama K, Karaki M, Goto R, Inamoto R, Samukawa Y (2015). Preference evaluation and perceived sensory comparison of fluticasone furoate and mometasone furoate intranasal sprays in allergic rhinitis. Auris Nasus Larynx.

[12] Meltzer E, Stahlman JE, Leflein J, Meltzer S, Lim J, Dalal AA (2008). Preferences of adult patients with allergic rhinitis for the sensory attributes of fluticasone furoate versus fluticasone propionate nasal sprays: a randomized, multicenter, double-blind, single-dose, crossover study. Clin Ther.

[13] Bernstein DI, Berkowitz RB, Chervinsky P, Dvorin DJ, Finn AF, Gross GN (1999). Dose-ranging study of a new steroid for asthma: mometasone furoate dry powder inhaler. Respir Med.

[14] Nayak AS, Banov C, Corren J, Feinstein BK, Floreani A, Friedman BF (2000). Once-daily mometasone furoate dry powder inhaler in the treatment of patients with persistent asthma. Ann Allergy Asthma Immunol.

[15] Noonan M, Karpel JP, Bensch GW, Ramsdell JW, Webb DR, Nolop KB (2001). Comparison of once-daily to twice-daily treatment with mometasone furoate dry powder inhaler. Ann Allergy Asthma Immunol.

[16] Kemp JP, Berkowitz RB, Miller SD, Murray JJ, Nolop K, Harrison JE (2000). Mometasone furoate administered once daily is as effective as twice-daily administration for treatment of mild-to-moderate persistent asthma. J Allergy Clin Immunol.

[17] Penagos M, Compalati E, Tarantini F, Baena-Cagnani CE, Passalacqua G, Canonica GW (2008). Efficacy of mometasone furoate nasal spray in the treatment of allergic rhinitis. Meta-analysis of randomized, double-blind, placebo-controlled, clinical trials. Allergy.

[18] Baldwin CM, Scott LJ (2008). Mometasone furoate: a review of its intranasal use in allergic rhinitis. Drugs.

[19] Blaiss MS (2008). Evolving paradigm in the management of allergic rhinitis associated ocular symptoms: intranasal corticosteroids. Curr Med Res Opin.

[20] Nielsen LP, Dahl R (2003). Comparison of intranasal corticosteroids and antihistamines in allergic rhinitis: a review of randomized, controlled trials. Am J Respir Med.

[21] Weiner JM, Abramson MJ, Puy RM (1998). Intranasal corticosteroids versus oral H1 receptor antagonists in allergic rhinitis: systematic review of randomised controlled trials. BMJ.

[22] de Moor C, Reardon G, McLaughlin J, Maiese EM, Navaratnam P (2012). A retrospective comparison of acute rhinosinusitis outcomes in patients prescribed antibiotics, mometasone furoate nasal spray, or both. Am J Rhinol Allergy.

[23] Stjärne S, Blomgren K, Cayé-Thomasen P, Salo S, Søderstrøm T (2006). The efficacy and safety of once-daily mometasone furoate nasal spray in nasal polyposis: a randomized, double-blind, placebo-controlled study. Acta Otolaryngol.

[24] Stjärne P, Mösges R, Jorissen M, Passàli D, Bellussi L, Staudinger H (2006). A randomized controlled trial of mometasone furoate nasal spray for the treatment of nasal polyposis. Arch Otolaryngol Head Neck Surg.

[25] Aboud SK, Husain S, Gendeh BS (2014). Comparison between endonasal endoscopic polyp size scores and quality of life outcome after optimal medical treatment. Rhinology.

[26] Gawchik S, Goldstein S, Prenner B, John A (2003). Relief of cough and nasal symptoms associated with allergic rhinitis by mometasone furoate nasal spray. Ann Allergy Asthma Immunol.

[27] Minshall E, Ghaffar O, Cameron L, O’Brien F, Quinn H, Rowe-Jones J (1998). Assessment by nasal biopsy of long-term use of mometasone furoate aqueous nasal spray (Nasonex) in the treatment of perennial rhinitis. Otolaryngol Head Neck Surg.

[28] Bhargava R, Chakravarti A (2014). Role of mometasone furoate aqueous nasal spray for management of adenoidal hypertrophy in children. J Laryngol Otol.

[29] Bhargava R, Chakravarti A (2014). A double-blind randomized placebo-controlled trial of topical intranasal mometasone furoate nasal spray in children of adenoidal hypertrophy with otitis media with effusion. Am J Otolaryngol.

[30] Rezende RM, Amato FS, Barbosa AP, Menezes UP, Rezende P, Ferriani VP (2015). Does atopy influence the effectiveness of treatment of adenoid hypertrophy with mometasone furoate?. Am J Rhinol Allergy.

[31] Rodríguez-Martínez CE, Sossa-Briceño MP, Vladimir Lemos E (2015). Cost-effectiveness analysis of mometasone furoate versus beclomethasone dipropionate for the treatment of pediatric allergic rhinitis in Colombia. Adv Ther.

[32] Chur V, Small CB, Stryszak P, Teper A (2013). Safety of mometasone furoate nasal spray in the treatment of nasal polyps in children. Pediatr Allergy Immunol.

[33] Affrime MB, Kosoglou T, Thonoor CM, Flannery BE, Herron JM (2000). Mometasone furoate has minimal effects on the hypothalamic-pituitary-adrenal axis when delivered at high doses. Chest.

[34] Skoner DP, Meltzer EO, Milgrom H, Stryszak P, Teper A, Staudinger H (2011). Effects of inhaled mometasone furoate on growth velocity and adrenal function: a placebo-controlled trial in children 4-9 years old with mild persistent asthma. J Asthma.

[35] Tan RA, Corren J (2008). Mometasone furoate in the management of asthma: a review. Ther Clin Risk Manag.

[36] Fausnight TB, Craig TJ (2011). Mometasone furoate dry powder inhaler for the treatment of asthma. Expert Opin Pharmacother.

[37] Milgrom H (2010). Mometasone furoate in children with mild to moderate persistent asthma: a review of the evidence. Paediatr Drugs.

[38] Sharpe M, Jarvis B (2001). Inhaled mometasone furoate: a review of its use in adults and adolescents with persistent asthma. Drugs.

[39] Samoliński B, Nowicka A, Wojas O, Lipiec A, Krzych-Fałta E, Tomaszewska A (2014). Intranasal glucocorticosteroids - not only in allergic rhinitis In the 40th anniversary of intranasal glucocorticosteroids’ introduction. Otolaryngol Pol.

